# Time Delay in Motor Vehicle Accident Arrival: A Critical Analysis of Trauma Team Activation

**DOI:** 10.7759/cureus.58070

**Published:** 2024-04-11

**Authors:** Zachary Taylor, Andrew McCague

**Affiliations:** 1 Medicine, College of Osteopathic Medicine of the Pacific - Northwest, Western University of Health Sciences, Lebanon, USA; 2 General Surgery, Desert Regional Medical Center, Palm Springs, USA; 3 Trauma and Acute Care Surgery, Desert Regional Medical Center, Palm Springs, USA

**Keywords:** trauma team activation criteria, overtriage, trauma team, trauma team activation, trauma centre

## Abstract

Introduction

This research aims to investigate the role of time since trauma (TST) in refining trauma team activation (TTA) criteria within a level I trauma center. We analyze the association between TST and post-emergency department (ED) disposition, proposing new insights for the enhancement of TTA criteria.

Methods

A retrospective analysis was conducted on a dataset comprising 3,693 patients presenting to a level I trauma center following motor vehicle accidents (MVAs) from 2016 to 2021. Data from a trauma registry, encompassing time of injury, time of ED arrival, TTA status, and post-ED disposition, were utilized. TST was calculated as the difference between the time of injury and the time of ED arrival. Patients that received TTA, full or partial, were categorized based on TST (less than one hour, one to two hours, and two or more hours). Statistical analyses, including chi-square tests, were performed using the Statistical Analysis System (SAS) (version 3.8, SAS Institute Inc., Cary, NC).

Results

Of the 1,261 patients meeting the criteria, 98.3% received TTA, with decreasing TTA rates observed with increasing TST (p = 0.0076). A significant association was found between TST and post-ED disposition for patients who received TTA (p = 0.0007). Compared to the other TST groups, a higher proportion of patients with a TST of two or more hours were admitted, sent to the intensive care unit (ICU), and sent to the operating room (OR).

Conclusion

The study indicates a statistically significant relationship between TST and TTA rates, challenging our assumptions about the decreased need for TTA over time. While a longer TST was associated with a lower percentage of TTA, patients with a TST of two or more hours demonstrated increased rates of admission, ICU utilization, and surgical interventions. This suggests that TTA criteria may benefit from refinement to include patients with longer TST. Acknowledging study limitations, such as a small sample size and retrospective design, this research contributes valuable insights into potential considerations for optimizing trauma care protocols.

## Introduction

A trauma team is a specialized, multidisciplinary group of healthcare professionals activated in response to patient presentations involving trauma or severe injuries. The primary goals of trauma team activation (TTA) are to rapidly assess and stabilize patients, address life-threatening injuries, initiate the necessary interventions to improve the chances of survival, and minimize long-term complications. The criteria for TTA varies between healthcare institutions based on multiple factors including resource availability, patient demographics, and specialized services available.

Although pivotal in providing quick, effective care, TTA is not without cost to both patients and institutions. On average, patient fees for full TTA range from $1,000 to $61,734, with a median fee of $9,500, while the average readiness costs for a level I and level II trauma center are $10,078,506 and $4,925,103, respectively [1,2]. Therefore, the approach to activating a trauma team should be a judicious one. While TTA is essential for addressing traumatic injuries and improving patient outcomes, unnecessary activations can contribute to increased healthcare costs. This can subsequently burden already ailed patients with additional expenses and financially overwhelm healthcare institutions. The criteria for TTA must be expansive enough to allow care for those who qualify, as patients who qualify may exhibit higher injury severity and mortality risks, but specific enough to avoid driving up unnecessary costs for those who may not require such services [3]. Institutions must consistently reevaluate and refine established TTA criteria to achieve this delicate balance. This paper investigates time since trauma (TST) as it relates to patient clinical outcomes, proposing it as a parameter that could contribute to refining TTA criteria.

## Materials and methods

We performed a retrospective analysis of patients who presented to our level I trauma center after a motor vehicle accident (MVA) from 2016 to 2021. The data were pulled from our ImageTrend trauma registry, which is updated by our certified trauma registrars. This de-identified dataset included time of injury, time of arrival to the emergency department (ED), TTA status, and post-ED disposition. Post-ED dispositions included transfer to a higher level of care, general admission to the hospital, home, intensive care unit (ICU), skilled nursing facility (SNF), operating room (OR), morgue, and leaving against medical advice (AMA). We took the difference between the time of arrival to the ED and the time of injury to calculate a new parameter, TST. Information from the calculation of TST allowed us to see how long after traumatic injury patients were evaluated in the ED. We created three distinct categories based on patients’ TST: less than one hour, one to two hours, and two or more hours. We divided all patients into two groups, those who received TTA, including full and partial TTA, and those who did not. The trauma team was activated upon realization that any of the criteria in Table 1 or Table 2 existed either upon arrival of the patient, notification by emergency medical services (EMS), or identification of meeting the criteria at any time since the traumatic event. A full trauma team response was defined as the arrival of an attending ED physician, a minimum of three trauma/ED nurses, respiratory therapy, radiology technician, OR charge nurse or designee, ICU clinical manager or designee, phlebotomist, house supervisor, and social worker within five minutes of patient arrival along with a trauma surgeon within 15 minutes of patient presentation. A partial trauma response was defined as the arrival of an attending ED physician, a minimum of two trauma/ED nurses, a respiratory therapist, a radiology technician, and a phlebotomist within five minutes and a trauma surgeon's arrival within 30 minutes.

**Table 1:** Full Trauma Team Activation Criteria at a Level I Trauma Center

Full Trauma Team Activation Criteria
Unstable airway, breathing, or circulation: Intubated patients, with respiratory compromise, and inadequate tissue perfusion, requires intravenous fluids, vasopressors, or blood products to maintain vital signs or administration of blood products from a referring facility to maintain vital signs.
Abnormal vital signs: Respiratory rate less than 10 or greater than 29, infant respiratory rate less than 20, pulse less than 50 or greater than 120, adult systolic blood pressure less than 90, geriatric (age 65 or above) systolic blood pressure less than 100, or pediatric systolic blood pressure less than 70.
Mechanism of injury: Open or depressed skull fracture, penetrating injuries to the head, neck, chest, abdomen, back, groin, buttocks, or burns of second or third degree involving greater than 20% body surface area or significant burns involving face or airway.
Neurological status change: Blunt head injury with a Glasgow Coma Scale less than or equal to 13, focal neurologic deficit, unequal pupils, or suspected spinal injury with neurologic deficits.
Anatomic findings/injuries: Chest wall instability or deformity, major traumatic amputation proximal to wrist or ankle, crushed, degloved, mangled, or pulseless extremity, crush injuries involving the chest, abdomen, or pelvis, suspected unstable pelvic fractures, two or more proximal long bone fractures, bleeding controlled by tourniquet with hemodynamic instability, or attending emergency department physician discretion.

**Table 2:** Partial Trauma Team Activation Criteria at a Level I Trauma Center

Partial Trauma Team Activation Criteria
Mechanism of injury: High-risk auto crash and/or intrusion, including roof; greater than 12 inches in occupant site; or greater than 18 inches in any site, ejection (partial or complete), death in the same passenger compartment, motorcycle crash greater than 20 mph with significant injury, pedestrian hit at greater than 20 mph, fall (adult) greater than 15 feet, fall from any height for a person age 65 years or older with suspected orthopedic or neurologic injury with anticoagulant/antiplatelet therapy, fall (pediatric) greater than 10 feet, fall (pediatric) greater than three times the child's height, hanging or gunshot wound to extremity and is hemodynamically stable.
Abnormal vital signs: Respiratory rate less than 10 or greater than 29, infant respiratory rate less than 20, pulse less than 50 or greater than 120, adult systolic blood pressure less than 90, geriatric (age 65 or above) systolic blood pressure less than 100, or pediatric systolic blood pressure less than 70.

We first used chi-square analysis to compare TST and TTA to determine if differences in TTA based on TST were statistically significant. We then performed another chi-square analysis comparing TST to post-ED disposition to find a statistically significant difference between the duration of TST and the ultimate clinical course of trauma patients. We included patients regardless of the mechanism of arrival to the ED (i.e., ambulance, helicopter, police, or private vehicles). We excluded patients who were transferred in from another facility and those with an unknown time of injury, as this parameter was critical to the calculation of TST. Statistical analysis was performed using the Statistical Analysis System (SAS) (version 3.8; SAS Institute Inc., Cary, NC). A P value of less than 0.05 was considered statistically significant.

## Results

Our dataset included 3,693 patients. After excluding patients with an unknown time of injury, 1,261 patients met the criteria for analysis. Of these 1,261 patients, 1,240 (98.3%) received TTA, and 21 (1.67%) did not. Although most patients in each group received TTA, we found that the total percentage of patients who received TTA decreased with increasing TST, with the largest drop-off between a TST of one to two hours and two or more hours. These findings were statistically significant, X^2 (degrees of freedom (DF) = 2, N = 1,261) = 9.7528, p = 0.0076. See chi-square analysis in Table 3.

**Table 3:** Chi-Square Test Comparing Trauma Team Activation Between Time Since Trauma Groups Data were presented as N (%).

Time since trauma	Trauma team activation	Total
Less than one hour	569 (45.12)	578 (45.84)
One to two hours	489 (38.78)	493 (39.1)
Two or more hours	182 (14.43)	190 (15.07)

We then decided to complete a chi-square analysis comparing TST and post-ED disposition in patients who received TTA. We found that a slight majority of patients with a TST of less than one hour and one to two hours were sent home after the ED, with the next most common disposition being admission. Conversely, most patients with a TST of two or more hours were admitted to the hospital, followed by disposition to home. Within the group of two or more hours, 41.8% of patients were admitted to the hospital, 24.2% were sent to the ICU, and 8.8% were sent to the OR. Proportionally, the two-or-more-hours group showed higher rates of these dispositions within the group than those observed within the other two groups. These results were statistically significant, X^2 (DF = 14, N = 1,240) = 37.0141, p = 0.0007. See Table 4.

**Table 4:** Chi-Square Test Comparing Post-Emergency Department Disposition Between Time Since Trauma Groups Data were presented as N (%).

Time since trauma	Transferred to a higher level of care	Admitted	Home	Intensive care unit (ICU)	Left against medical advice (AMA)	Morgue	Operating room (OR)	Skilled nursing facility (SNF)	Total
Less than one hour	7 (0.56)	191 (15.4)	241 (19.44)	81 (6.53)	11 (0.89)	6 (0.48)	31 (2.5)	1 (.08)	569 (45.89)
One to two hours	8 (0.56)	167 (13.47)	205 (16.53)	76 (6.13)	4 (0.32)	1 (.08)	29 (2.34)	0	489 (39.44)
Two or more hours	1 (.08)	76 (6.13)	44 (3.55)	44 (3.55)	0	1 (.08)	16 (1.29)	0	182 (14.68)

These results suggest that patients arriving at the ED with a TST of two or more hours accounted for a lower percentage of patients who received TTA; however, the patients in this group showed higher proportions of more complex clinical trajectories than patients with a shorter TST.

## Discussion

The implementation of dedicated trauma teams in trauma centers has consistently demonstrated positive effects on the efficiency of care delivery and patient outcomes. A study at a level I trauma center assessed the effects of a full-time trauma service, featuring a 24-hour in-house attending, dedicated trauma admitting unit, regular trauma core curriculum, and multidisciplinary quality assurance meetings. Results indicated significant improvements in the average time spent in the ED for patients requiring operative intervention, intensive care unit admission, and observation ward admission. Additionally, TTA was associated with a notable reduction in trauma center closure hours due to ED overcrowding, emphasizing the positive impact on system-wide responsiveness. Notably, analyses controlling for demographic and injury-related variables revealed a 31% decrease in overall odds of death and a 42% decrease in odds of death among patients with severe head injuries after trauma team implementation [4]. Another investigation, conducted at a level I regional trauma center, focused on comparing outcomes for trauma patients with an injury severity score (ISS) greater than 12 based on TTA or service-by-service management. The study demonstrated statistically significantly better outcomes for the TTA group, indicating that the presence of a trauma team during resuscitative care leads to improved patient results. Utilizing the trauma score and injury severity score (TRISS) methodology and logistic regression equations, the TTA group consistently exhibited higher Z scores, supporting the conclusion that trauma team involvement is associated with superior outcomes [5]. Furthermore, a study of a trauma team incorporating full-time emergency medicine (EM)-trained physicians and trauma specialists showed significant improvements in mortality rates. This study revealed a reduction in overall mortality rates, particularly among patients with the most severe injuries. This substantial reduction in mortality rates after the implementation of a dedicated trauma team highlighted the critical role of trauma teams in enhancing patient outcomes [6]. A tertiary referral trauma center in Hong Kong also evaluated the association between trauma team activation protocols and patient survival. The results emphasized the importance of adherence to TTA protocols, as undercalls (failure to activate trauma teams when criteria were met) were associated with decreased survival, particularly in patients with a moderately poor probability of survival. Although TTA itself does not guarantee better survival, adherence to established protocols was shown to optimize processes of care and potentially lead to improved patient outcomes. These findings collectively underscore the multifaceted benefits of trauma teams, ranging from enhanced timeliness of care to significant improvements in patient survival rates [7]. Beyond the primary survival benefits for severely injured patients, trauma center designation and the associated trauma teams have shown a "halo effect," even extending positive outcomes to patients experiencing non-traumatic hemorrhage [8]. These findings collectively emphasize the multifaceted advantages of trauma teams, showcasing their effectiveness not only in traumatic injuries but also in diverse and emergent clinical scenarios.

Despite the effectiveness of trauma teams, the cost landscape of trauma care is intricate. Trauma care expenses encompass both ongoing readiness costs incurred by trauma centers and the more specific trauma team response fees (TTRF). In a comprehensive study exploring trauma center readiness costs, expenses were categorized into administrative/program support staff, clinical medical staff, in-house operating room, and education/outreach. Clinical medical staff emerged as the costliest component, constituting 55% and 64% of costs for level I and level II trauma centers, respectively. The study underscores the significant financial burden on trauma centers and advocates for additional funding to meet the standards set by the American College of Surgeons [2]. Another aspect of trauma care costs involves the one-time TTRF, contributing to the overall financial burden on patients. A nationwide survey of 525 trauma centers aimed to understand the determination process and variations in TTRF amounts. However, only 8.8% of the surveyed trauma centers shared their scheduled fees, revealing the elusive nature of this aspect of trauma care costs. The lack of standardized processes and insufficient data make it challenging to grasp the true costs associated with TTA. Trauma centers struggle to maintain financial viability amid increasing governmental and organizational regulations, emphasizing the need for collaboration between trauma centers and regulatory agencies to strike a balance between quality trauma care and justified charges [1].

Examining trauma activation fees across the United States, a cross-sectional study identified substantial variability among hospitals. Trauma activation fees, both tier 1 (full activation) and tier 2 (partial activation), exhibited a wide range, with the median tier 1 fee being $9,500 and the mean reaching $13,349. Regional variations were pronounced, with hospitals in the West, particularly in California, charging substantially higher fees. Additionally, for-profit hospitals, notably those owned by the HCA Healthcare system, imposed higher fees than other types of hospitals. The study's findings highlight the disparate financial implications for patients with serious traumatic injuries based on the trauma center they are brought to, prompting a call for the standardization of trauma activation fees to ensure equitable and transparent cost structures. Inferences from these studies collectively emphasize the intricate financial challenges faced by trauma centers and patients, stressing the need for comprehensive understanding, transparent practices, and collaborative efforts to ensure optimal trauma care without compromising financial sustainability [9].

Moreover, the financial challenges faced by trauma centers are further exacerbated by the phenomenon of overtriage, which involves the unnecessary activation of trauma teams for patients who do not require the full spectrum of trauma care. Overtriage has been shown to significantly increase the costs of trauma care, as it leads to the allocation of resources and personnel to situations that may not warrant such intensive intervention [10]. Unnecessary TTA can strain the already limited resources of trauma centers, contributing to heightened expenses without corresponding benefits for patients. Addressing overtriage becomes crucial for optimizing resource utilization and mitigating the economic burden on trauma centers and patients. A concerted effort towards refining TTA criteria and ensuring their precision is imperative to strike a balance between providing quality care and maintaining financial sustainability for trauma centers. In summary, the intricate financial landscape of trauma care necessitates a holistic approach, encompassing readiness costs, team response fees, regional variations, and the critical consideration of the impact of overtriage on costs.

With the necessary fluidity of TTA criteria in mind, our study offers insight into the use of TST as a factor to consider when refining TTA criteria. As the criteria for our level I trauma center currently stands, the trauma team is activated if a patient meets any of the criteria at any time before or upon arrival to the ED. This could potentially lead to overtriage of patients who met a criterion at the time of injury, but stabilized and did not meet any criteria by the time of arrival. One of the goals of calculating TST was to show the variation in how much time passes between a traumatic event or injury and a patient’s arrival at the ED. This variety in duration calls for investigation into how it relates to TTA criteria and a patient’s clinical course. Our initial hypothesis was that patients with a longer TST would be more stable by the time of arrival to the ED, thus nullifying the need for TTA.

Upon analysis of the dataset, we found a statistically significantly higher percentage of patients with a TST of less than one and two hours received TTA than those presenting with a TST of two or more hours. This finding may indicate that delayed presentations are associated with fewer qualifications for TTA. Despite patients with a TST of one or more hours accounting for a lower percentage of TTA, we did find that a significantly higher proportion of patients within this group were admitted and sent to the ICU and to the OR, possibly indicating a more complicated clinical course for these patients compared to those with a shorter TST. Ultimately, our study showed that patients who presented to the ED with a TST of two or more hours were responsible for a lower percentage of TTA, but were proportionally more likely to have a clinical course that required general admission, intensive care, or surgery. This finding suggests that a longer TST does not necessarily indicate greater patient stability or less need for TTA. Our data show that refining TTA criteria to include more patients with a longer TST may be beneficial. Future research should investigate the specific criteria met by patients within each TST group to examine patterns that may reveal which criteria are more likely present or absent with delayed presentations.

While there is limited research regarding the use of TST in TTA criteria refinement, many studies have explored different ways to address the challenges associated with non-specific TTA criteria. Several investigations into the impact of changes in TTA protocols, such as transitioning to a two-tier system, are ongoing. A prospective study assessed the impact of a two-tiered trauma response protocol on the expediency of trauma patient identification, evaluation, and treatment in the ED. The implementation of this protocol significantly decreased ED length of stay, allowing for more rapid identification and management of trauma patients requiring hospitalization. The two-tiered approach facilitated accurate identification of the most seriously injured patients, optimizing the allocation of resources and improving overall efficiency [11]. A two-tiered TTA protocol was also shown to be associated with reduced undertriage, but increased overtriage [12]. On the other hand, a shift to a one-tier system showed increased undertriage and overall mortality, emphasizing the importance of carefully considering TTA protocol changes to avoid compromising patient care [13]. Additionally, studies conducted in trauma centers have implemented changes to TTA protocols to improve accuracy and resource utilization. For instance, a Canadian level I trauma center implemented a quality improvement project to enhance TTA compliance, achieving an improvement from 58.8% to over 90%. This success highlights the benefits of a well-defined quality-improvement process in achieving sustained improvements in TTA compliance and subsequent trauma care processes [14].

One international survey, involving 37 countries, sought to establish post hoc criteria for TTA, revealing a high agreement rate of over 75% for 12 out of 20 proposed criteria. This suggests the potential for universally applicable TTA criteria, regardless of a country's income level. However, the study emphasizes the need for ongoing international discussions to achieve a consensus on a universal set of criteria for quality assessment and validation of field triage protocols [15].

Future research in TTA and associated criteria presents a dynamic landscape with several key areas deserving attention. A primary focus should be on the standardization and validation of criteria, urging multi-center collaborations to establish universally accepted standards adaptable to diverse trauma settings. Mechanism criteria also demand further refinement to enhance specificity without sacrificing sensitivity, potentially leveraging advanced technologies such as machine learning algorithms for more precise interpretation. Unraveling the impact of TTA on patient outcomes, especially through prospective studies and long-term follow-ups, is crucial for establishing a direct correlation between prompt activation and improved clinical results. The financial implications of TTA underscored in the literature call for cost-effectiveness analyses considering direct costs, long-term healthcare utilization, and societal impacts. Integration of advanced technologies, such as real-time data analytics and telemedicine, into TTA criteria, offers promising avenues for improved decision-making. Pediatric-specific TTA criteria need dedicated exploration, considering age-specific parameters and challenges associated with pediatric trauma care. Furthermore, research should delve into the perspectives of patients and caregivers, employing qualitative methods to inform patient-centered approaches to TTA. International collaboration and comparison studies are imperative to understand and adapt TTA criteria across different healthcare systems, promoting cross-cultural learning and identification of best practices. Addressing these diverse research pathways will contribute significantly to the ongoing evolution of trauma care protocols, ensuring efficiency, and patient-centeredness. Interdisciplinary collaboration and advancements in technology will be pivotal in shaping the trajectory of future research in this critical domain.

Limitations

It is crucial to acknowledge our relatively small sample size and several limitations inherent in our study design. The retrospective nature of the study introduces potential biases, including selection bias and the reliance on pre-existing data. Being a single-center study conducted at a level I trauma center may limit the generalizability of findings to broader healthcare settings with different protocols and patient populations. The use of a deidentified dataset raises concerns about the loss of critical patient-specific details that could influence outcomes, and the exclusion of patients with an unknown time of injury may introduce selection bias. The specific criteria for TTA, both full and partial responses, are center-specific, restricting the external validity of the study. Furthermore, the accuracy of the calculated TST depends on the precision of recorded times, and variations may impact the validity of TST as an independent variable.

## Conclusions

The exploration of dedicated trauma teams within trauma centers has consistently revealed positive impacts on care efficiency and patient outcomes. A comprehensive review of studies highlights the benefits, ranging from decreased ED times to significant reductions in mortality rates, emphasizing the critical role of specialized teams. However, the intricate financial landscape of trauma care poses substantial challenges to trauma centers. Variability in trauma activation fees, coupled with the financial strain induced by overtriage, underscores the need for a holistic approach to financial sustainability and TTA criteria refinement. Our study introduces the concept of TST to refine TTA criteria, revealing the importance of considering time variations in patient presentations. While acknowledging limitations, the study suggests that a longer TST does not necessarily negate TTA need and may even be associated with a more complex clinical course.

## References

[1] Zitek T, Pagano K, Mechanic OJ, Farcy DA (2023). Assessment of trauma team activation fees by US region and hospital ownership. JAMA Netw Open.

[2] Ashley DW, Mullins RF, Dente CJ (2019). How much green does it take to be orange? Determining the cost associated with trauma center readiness. J Trauma Acute Care Surg.

[3] Chien DS, Yiang GT, Liu CY (2022). Association of in-hospital mortality and trauma team activation: a 10-year study. Diagnostics (Basel).

[4] Cornwell EE, Chang DC, Phillips J, Campbell KA (2003). Enhanced trauma program commitment at a level I trauma center: effect on the process and outcome of care. Arch Surg.

[5] Petrie D, Lane P, Stewart TC (1996). An evaluation of patient outcomes comparing trauma team activated versus trauma team not activated using TRISS analysis. J Trauma.

[6] Gerardo CJ, Glickman SW, Vaslef SN, Chandra A, Pietrobon R, Cairns CB (2011). The rapid impact on mortality rates of a dedicated care team including trauma and emergency physicians at an academic medical center. J Emerg Med.

[7] Rainer TH, Cheung NK, Yeung JH, Graham CA (2007). Do trauma teams make a difference? A single centre registry study. Resuscitation.

[8] Vuong P, Sample J, Zimmermann ME, Saldinger P (2017). Trauma team activation: not just for trauma patients. J Emerg Trauma Shock.

[9] Neeki MM, Serrano J, Dong F (2022). Variation in trauma team response fees in United States trauma centers: an additional undisclosed variable cost in trauma care. Cureus.

[10] Dowd MD, McAneney C, Lacher M, Ruddy RM (2000). Maximizing the sensitivity and specificity of pediatric trauma team activation criteria. Acad Emerg Med.

[11] Tinkoff GH, O'Connor RE, Fulda GJ (1996). Impact of a two-tiered trauma response in the emergency department: promoting efficient resource utilization. J Trauma.

[12] Rehn M, Lossius HM, Tjosevik KE, Vetrhus M, Østebø O, Eken T (2012). Efficacy of a two-tiered trauma team activation protocol in a Norwegian trauma centre. Br J Surg.

[13] Thorsen K, Narvestad JK, Tjosevik KE, Larsen JW, Søreide K (2022). Changing from a two-tiered to a one-tiered trauma team activation protocol: a before-after observational cohort study investigating the clinical impact of undertriage. Eur J Trauma Emerg Surg.

[14] Verhoeff K, Saybel R, Fawcett V, Tsang B, Mathura P, Widder S (2019). A quality-improvement approach to effective trauma team activation. Can J Surg.

[15] Waydhas C, Trentzsch H, Hardcastle TC, Jensen KO (2021). Survey on worldwide trauma team activation requirement. Eur J Trauma Emerg Surg.

